# Vitrectomy in full thickness macular holes on top of a pigment epithelial detachment in age-related macular degeneration (AMD). Surgical consideration and review of the literature

**DOI:** 10.1016/j.ajoc.2021.101154

**Published:** 2021-07-10

**Authors:** Paula S. Meyer, Marc T. Kammann, Carsten H. Meyer

**Affiliations:** Macular Center Davos, Switzerland

**Keywords:** Vitreomacular traction, Macular hole, Pigment epithelial detachment, Age-related macular degeneration, Vitreoretinal surgery, Epiretinal membrane

## Abstract

**Purpose:**

To present the surgical treatment of a full thickness macular hole (MH) caused by a vitreomacular traction (VMT) on top of an adjacent subfoveal pigment epithelial detachment (PED) in age-related macular degeneration (AMD).

**Observation:**

A 77-year-old female with a subfoveal PED receiving consecutive intravitreal injections noticed a sudden decreased visual acuity (VA) due to the development an occult MH in her right eye after 19 repeated intravitreal anti vascular endothelial growth factor (VEGF)-injections. Her initial VA declined from 20/50 to 20/400. The firm VMT induced a rupture of the multi-layered retina and may progress to an RPE-tear or possible to a subretinal haemorrhage. We discussed with the patient the risks of the natural progression and explained possible treatment options: We continued her anti-VEGF combined with air bubble injections to induce a posterior vitreous detachment, to stabilise the retinal architecture, reduce the subretinal fluid and avoid possible intraoperative bleeding. As injections did release the VMT, vitrectomy released the posterior vitreous from the optic nerve and trimmed it towards the central retina. Peeling with brilliant blue removed the internal limiting membrane without any signs of bleeding, rupture of the PED or enlargement of the MH, prior to the installation of 10% SF6 gas. The postoperative optical coherence tomography (OCT) on day 5 confirmed a closed MH, while the size, shape and pattern of the PED remained unchanged. Her VA increased from 20/400 to 20/50 (equal to her previous VA prior to the MH-formation). To avoid a potential progression of the PED, we maintained her retreatment intervals at 5 weeks for the next 6 months. A literature review presents similar intraoperative approaches and postoperative outcomes in 8 out of the 9 published cases.

**Conclusions and importance:**

VMT can induce an occult MH on top of a PED, causing a significant loss of vision. When gas injections are not successful, surgery may release the traction, restore the retinal architecture, and significantly improve and maintain the VA over a documented long-term observation. The epiretinal procedure should be assisted under regular anti-VEGF injections to maintain the subretinal architecture.

## 1 Introduction

Vitreomacular traction (VMT) may occur during aging from incomplete posterior vitreous detachment (PVD) with remaining adhesions at the central macula, leading to the formation of a macular hole (MH) or cystoid oedema.^1^ While VMT has also been observed in conjunction with different stages of age-related macular degeneration (AMD),^2^ it may have an impact on the progression from non-to exudative AMD in selected patients.^3^ Many eyes with incomplete PVD present particularly firm vitreopapillary adhesions at the optic nerve head and central fovea^4^; thus, any contraction between these two firm adhesions may induce a vector of share traction from the fovea towards the optic nerve head, explaining why many pigment epithelial detachments (PED) typically rupture at their temporal edge towards the centre.^5^

However, the concurrent occurrence of a VMT adjacent to a PED can also lead to full thickness MH-formation in rare cases. The coincidental development of such an occult MH on top of a PED will lead to unexplained vision loss. Previously, we described this phenomenon as a natural cause^6^ or after consecutive intravitreal injections.^7^ While small idiopathic MH may close spontaneously,^9^ their presence on top of a PED in conjunction with very firm VMT appears to persist predominantly, possibly progressing to a secondary subretinal (SR) pigment epithelial (RPE)-tear^8^ or SR haemorrhage.

Here, we demonstrate the surgical release of a persisting VMT in an MH over a subfoveal PED to discuss possible considerations in the preoperative preparation and intraoperative approach of our case, and to compare our postoperative outcome with the few published cases of this rare entity and approach.

## 2 Case report

A 77-year-old female with neovascular AMD and chronic PED OD had been treated with 19 intravitreal anti-vascular endothelial growth factor (VEGF)-injections over the course of 3 years. On optical coherence tomography (OCT) there was a significant PED with some SR fluid prior to each injection. Over time, we noticed an occult VMT at the foveal centre, which became evident when the retinal was thin soon after each anti-VEGF injection. Her visual acuity (VA) remained stable at 20/50 under this treatment regime, until she presented a sudden decrease of VA in her right eye from 20/400. On dilated fundus biomicroscopy, as well as standard OCT-scanning, no AMD-progression was apparent. A meticulous thin lined en face OCT-examination revealed an unchanged pattern of the PED, while a MH with a VMT on top of the PED became visible (Fig. 1a). This tiny but full thickness occult MH with a diameter of 568 μm may have been responsible for the decreased VA, so we discussed further treatment options with our patient. The presence of the firm VMT had already induced the rupture of the multi-layered retina; thus, an additional rupture of the PED monolayer of the RPE may progress to an RPE-tear and possible SR haemorrhage with a consecutive loss of the remaining VA. We discussed the risks of natural progression with the patient and explained possible treatment options: At first, we combined the next intravitreal anti-VEGF injections with a consecutive 1 cc air bubble application and asked the patient to maintain in a prone position over night, but no VMT release was observed. She refused larger C3F8 gas injection, possibly inducing an increased intraocular pressure, as she lives at the altitude above 2400 m in the Swiss alps. We discussed possible enzymatic release of the posterior vitreous by an ocriplasmin injection,^14^ although the enzyme may also affect the RPE tight junctions inducing SR bleedings or a RPE rupture. We also reported her about our previous successful experience with a plana pars vitrectomy (PPV) with a plane release of the VMT to close the MH in a similar case,^7^ although any surgical manipulation may also enhance the traction on the fovea, possibly leading to an iatrogenic RPE-tear or consecutive SR haemorrhage.

**Fig. 1a:**
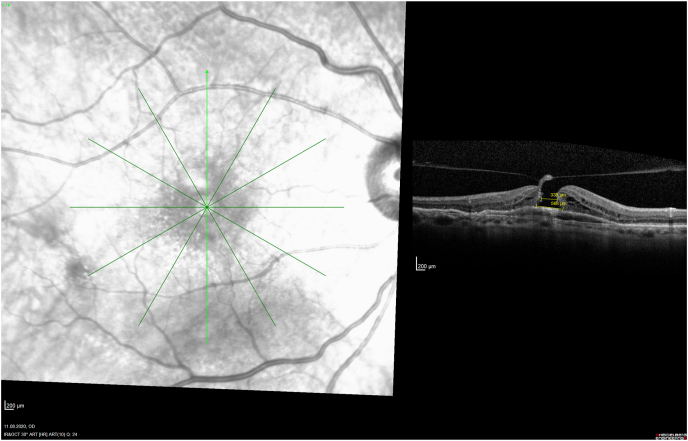
Preoperative OCT, right fundus: The vertical OCT examination demonstrates a full thickness macular hole (diameter 568 μm) overlying a pigment epithelial detachment.

After extensive discussion with the patient, she agreed to continue with her anti-VEGF injections until we observed a shallow PED and compact neuroretina around the MH. The goal of these injections was to **A)** to stabilise the retinal architecture, **B)** reduce the SR fluid and **C)** seal the CNV of the PED as best as possible to avoid further bleeding. Delayed chromovitrectomy was now performed under general anaesthesia with initial staining of the vitreous with triamcinolone. With a high cutting rate of 5000 p.m. and a gentle aspiration of 40 mmHg, we removed the core of the vitreous under slow movements approaching the retinal surface. After the release of the vitreous from the optic nerve head, we gently trimmed the posterior vitreous towards the central retina and further to the periphery without any signs of a bleeding, rupture of the PED or enlargement of the MH. With the uncomplicated progress of the surgery, we even decided to stain the internal limiting membrane (ILM) with brilliant blue and performed a gentile ILM-peeling from the periphery towards the macular hole. The release of the ILM was gentle and with no significant tract; thus, we closed the surgery with a conventional fluid gas exchange and installation of 10% SF6 gas. The patient was asked to keep a prone position over night and was examined during the next few days. The intraocular gas bubble vanished over 5 days and her intraocular pressure remained normal at 18 mmHg. The OCT on her 5th postoperative day revealed a closed MH, while the size, shape and pattern of the PED had not changed (Fig. 1b). During the 6-month follow-up period, her right eye developed no RPE-tear formation of the SR haemorrhage. To avoid progression of the PED and with respect of the altered pharmacokinetics of the anti-VEGF drug in the vitrectomized vitreous cavity, we maintained her retreatment intervals at 5 weeks for the next 6 months. Her VA increased from 20/400 to 20/50 (equal to her previous VA prior to the MH-formation and she was able to read again).

**Fig. 1b:**
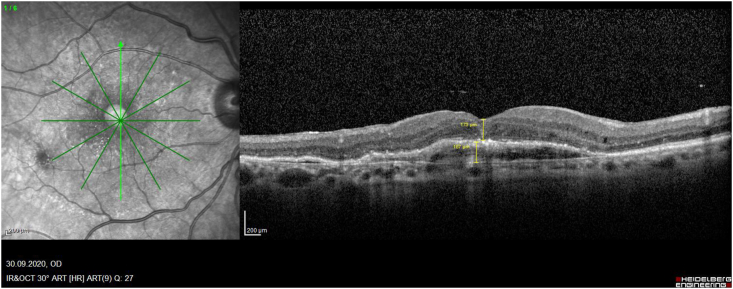
Postoperative OCT, right fundus: One week after vitrectomy with ILM-peeling and gas application, the OCT examination demonstrates closure of the macular hole, and complete restauration of the anatomical architecture (173 μm thickness) with a physiological foveal depression, while the persisting subretinal PED (165 μm) remained unchanged.

## 3 Discussion

The aging vitreous may play an important role as a coincident factor in the pathogenesis of numerous macular diseases.^1^ While VMT is a potential risk for exudative AMD,^10^^,^^11^ it may also reduce the efficacy of anti-VEGF injections, requiring more frequent retreatments during the extension period.^12^ The higher age of the elderly patient^13^ and the architecture of the vitreoretinal interface^14^ showed signs of firm adhesions with a low probability of spontaneous separation, considering a surgical release in this case. The concurrent presence of MH and CNV has been evaluated in the past. In 2001, *Elsing* et al. reviewed the surgical outcome of such cases with 20-gauge instruments by combined MH-repair and CNV-removal,^15^ and reported a final VA, ranging from 20/100 to hand motions, limited by the cumulative retinal and RPE damage. More recently, *Hirata* et al. reported the removal of a CNV-membrane with 25-gauge instruments through the coincident presence of a macular hole after anti-VEGF therapy for AMD and observed a rapid resolution of the intraretinal macular oedema with a fast anatomical and functional recovery to 20/40.^16^

The development of MH after intravitreal injections has been described in the past in eyes with the primary diagnosis of neovascular AMD, diabetic macular oedema, branch retinal vein occlusion or vasoproliferative tumours.^17^ In addition to their primary vascular exudation, most eyes present signs of epiretinal traction or VMT, predisposing tangential or anteroposterior traction after each injection.^18^, ^19^, ^20^, ^21^, ^22^, ^23^ Several epiretinal or SR mechanisms may trigger the formation of MH after intravitreal injections: first, the applied volume of fluid and its chemical compounds modify and liquefy the structure of the vitreous gel (syneresis),^24^ leading to incomplete PVD and anteroposterior traction on the VMT. Second, *Grigoropoulos* et al. hypothesised that the mechanical insertion of the injection needle leads to a globe deformation and retinal traction, while the removal of the needle may cause a consecutive vitreous micro-incarceration exacerbating the underlying VMT.^25^ Previously, we also analysed and calculated the initial release velocity of an injected Ozurdex implant by a high-speed camera and determined that the dragging force of the vitreous gel decelerates the implant, thus avoiding damage to the retinal surface.^26^ In addition, the contraction of the CNV complex and flattening of the PED may create an anterior-posterior vectorial force diametrically opposite to the direction of the overlying VMT,^5^ thus increasing the risk for a retinal defect, and contributing to formation of a MH. Finally, the aim of the potent anti-VEGF drug is to modulate the activity of the CNV, inducing a rapid contraction of the vasoproliferative membrane with forces on the CNV and PED, with tangential traction on the overlaying retina. Combined MH and RPE-tear may therefore both rely on a contraction of the RPE as the exacerbating factor.

Our case also presented the simultaneous presence of two separate pathologies below and above the retina, requiring additional well-considered proceedings:

I. The chronic underlying SR PED must be sufficiently treated by ant-VEGF injections. An active lesion may potentially cause SR bleeds, during either the surgical manipulation of the adjacent epiretinal VMT or the postoperative period. An undertreated PED prior to surgery may also cause significant SR fluid accumulation, making the neuroretina less compact and resistant to the presumed tractional shear force during the epiretinal ILM-peeling.
II. Any systemic aspirin or warfarin application should be interrupted, if possible, prior to and shortly after the surgery.
III. The stepwise intraoperative release of the epiretinal traction needs to be well-thought out and structured ahead of surgery:
  - The vitreous removal on top of such a delicate lesion requires a gentle and slow intentional removal of the vitreous with a high cutting rate and low aspiration, diminishing any possible traction on the perpendicular retinal lesion as much as possible.
  - Chromovitrectomy with triamcinolone may help to stain and visualise the posterior hyaloid for its safe and reliable removal.
  - The release of the posterior hyaloid should not start with the VMT itself, as unexpected firm adhesions may induce traction and vigorous force of the PED, possibly inducing a rupture of the thin RPE-monolayer or adjacent choroidal vessels causing a vision threatening subretinal haemorrhage. We recommend initiating the induction of PVD at first over the optic nerve head to release the vitreopapillary adhesion followed by trimming the posterior hyaloid only partially over the macular area.
  - Another consideration prior to surgery may be pneumatic vitreolysis by the intravitreal application of C3F8 gas to relieve vitreous traction and possibly close the macular hole.
  - Finally, we discussed whether a plain PVD may be enough to seal the MH on top of the PED, as many small conventional MHs close after a solely PPV without an ILM-peel.

However, we decided to intraoperatively stain and visualise the ILM with the application of brilliant blue by chromovitrectomy. We observed a well-delimited ILM and started an attempt to remove it at the outer macular area close to the vascular arcade, where the retina was not perpendicular to the fragile PED. We noticed a well-controlled, gentile release of the ILM and decided to extend the peeling in a slow manner from the periphery of the outer macula towards the rim of the MH. This manoeuvre should be avoided if the ILM presents firm adhesions, as small MH frequently close after the release of the VMT. The eye was closed after a conventional fluid-air-exchange and a fast conventional closure of the small MH presented within days. Once the epiretinal pathology has been treated, continuous retreatments of the subretinal PED with consecutive monthly anti-VEGF injections are recommend, as the pharmacokinetic durability of the fluid anti-VEGF drug may be reduced after removal of the vitreous according to previous measurements.^27^ On the one hand may the release of the vitreous traction reduce the activity of the CNV and frequency of consecutive injections,^11^ on the other hand we may face a shortened durability of the liquid drug in any vitrectomized vitreous cavity.^27^ Finally, in any eyes with the unexplained onset of new symptoms, we recommend extending the conventional 12-line radial OCT-scan to a raster en face OCT-examination to detect tiny occult lesions, which may have remained undetected with conventional OCT-protocols.^28^

While several papers described the rare development of MH after intravitreal injections in greater detail postulating the underling pathomechanism,^18^, ^19^, ^20^, ^21^, ^22^, ^23^ only 9 reported eyes have been treated by PPV (Table 1): One of these case reports was previously published by us^7^: This was a 67-year-old female with a subfoveal PED and adjacent occult CNV. During the full up-loading phase by three uneventful intravitreal ranibizumab injections, she developed an accelerating VMT and finally an MH on top of the PED. The vitreous traction was released during PPV and ILM-peeling, and her hole closed postoperatively, while the PED remained consistent. Today, with a documented 10-year follow-up we can address also important additional information regarding the long-term prognosis for the discussion of these rare eyes: The analysis of our consecutive OCT images (Fig. 2a, Fig. 2b, Fig. 2c, Fig. 2da–d) between our ppv in October 2009 and the latest follow-up in April 2021 shows a gradual anatomical closure of the macular hole over 4 years postoperative period (Fig. 2c), while the macular hole did not reopen at any time. In addition, the hights of the measured subretinal PED maintained constant over the next 6 years (Fig. 2d) and was controlled by repeated intravitreal injections. The annual frequency of the applied intravitreal injections did not differ in the previously non-vitrectomized PED with the VMT (n = 6 per year) prior to the vitrectomy, compared to the here vitrectomized eye with a PED after the released VMT (n = 5–6 injections per year). The measured retinal thickness remained stable under anti-VEGF therapy. However, we observed a constant progression of drusen associated with a gradual expanding area of the PED, corresponding with a progression of the underlying disease, while the hights of the PED itself gradually declined under the anti-VEGF therapy (Fig. 2e). The RPE layer of the PED maintained intact, no bleedings or RPE-tear occurred during the documented 10-year follow-up, while her postoperative VA declined by one line during this period.

**Table 1:** Treatment of macular holes after intravitreal injections with consecutive epiretinal traction by vitreoretinal surgery.

eyes	reference	patient age, gender ocular diagnosis	Previous treatment	VA preop (Snellen)	VA postop (Snellen)	macular hole (stage)	surgery	complication	follow-up (months)
1	Sato A. et al. 2020^29^	64-years, male OD, VMT, SRF, vasoproliverative Tx,	2x IVB	20/25	20/63	IV	phaco, 25g ppv, ILM peeling, 25% SF6	closed, no hem	12
**2**	Mukherjee C. et al. 2015^30^	81-years, male OD, VMT, PED	3x IVR	6/30	6/24	III	phaco, macular hole surgery	na	na
**3**		62-years, female OS, VMT, CNV	3x IVR	6/24	6/36	III	phaco, macular hole surgery	na	na
**4**	Lee YJ, Kim K. 2019^31^	56-years, male OD, ERM, DME	1x IVB	20/200	20/100	IV	phaco, 25g ppv, ILM peeling, 20% SF6	closed, serous RD, no hem	6
**5**	Muramatsu D. et al. 2015^32^	63-years, male OD, ERM, BRVO, SRF,	IVR	20/200	20/40	IV	phaco, 25g ppv, ILM peeling, 20% SF6	Closed no hem	5
**6**	Shif OA, Katz MSJ. 2018^33^	77-years, male OD, RPE-tear	IVR				phaco, 25g ppv, ILM peeling, 16% C3F8	na	na
**7**	Raiji VR, Eliott D, Sadda SR. 2013^34^	69-years, female OS, VMT, PED	IVR	20/200	20/60	IV	ppv, ILM peeling, gas	no hem	
**8**	Regatieri CV, Ducker JS. 2012^35^	76-years, male OD, VMT, CNV	1x IVR		not improved		ppv, ILM peeling, gas	open no hem	
**9**	Clemens C, Holz FG, Meyer CH. 2010^7^	67-years, female OS, VMT, PED	3x IVR	20/30	20/30	III	23g ppv, ILM peeling, 20% SF6	closed, no hem foveal lucency	12
**10**	Meyer PS, Kammann MT, Meyer CH. 2021	77-years, female OD, VMT, PED	25x IVR	20/200	20/50	III	23g ppv, ILM peeling, 20% SF6	closed, no hem	8

**Abbreviations**: **OD** oculus dexter, **OS** oculus sinister, **VA** visual acuity, **VMT** vitreomacular traction, **Tx** tumor, **SRF** subretinal fluid, **PED** pigment epithelial detachment, **CNV** choroidal neovascularization, **BRVO** branch retinal vein occlusion, **DME** diabetic macular oedema, **RPE** retinal pigment epithelium, **IVB** intravitreal bevacizumab, **IVR** intravitreal ranibizumab, **ILM** inner limiting membrane, **ppv** pars plana vitrectomy, **phaco** phacoemulsification, **SF** sulfur hexafluoride, **hem** haemorrhage.

**Fig. 2a:**
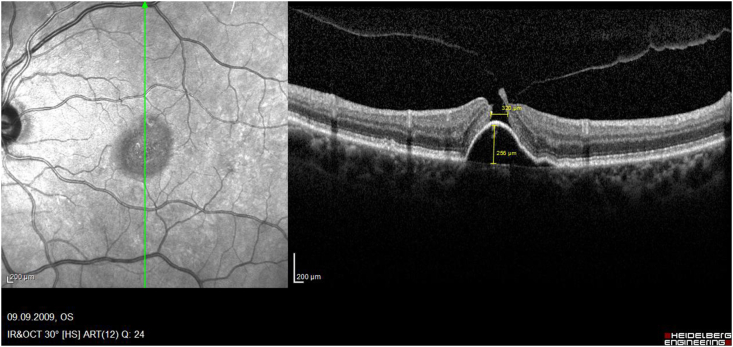
Historic 10 years long-term vertical OCT-scans of a closed macular hole on top of a PED after vitrectomy: A preoperative status of a PED with a height of 256 μm with a perpendicular macular hole with an aperture of 320 μm and a vitreomacular traction.

**Fig. 2b:**
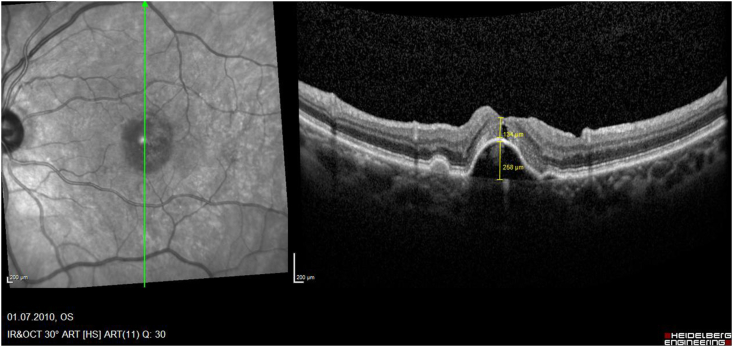
Six months postoperative status of a PED with an unchanged height of 258 μm, an outer retinal closure of the macular hole, retinal thickness 134 μm and a vitreomacular traction.

**Fig. 2c:**
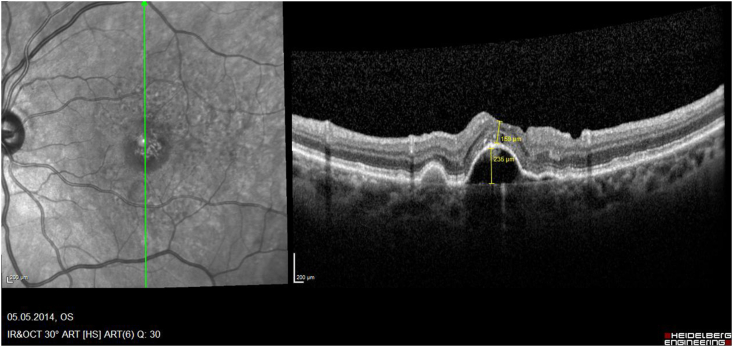
Four years postoperative status of a PED with a similar height of 235 μm, an outer retinal closure of the macular hole, retinal thickness 159 μm.

**Fig. 2d:**
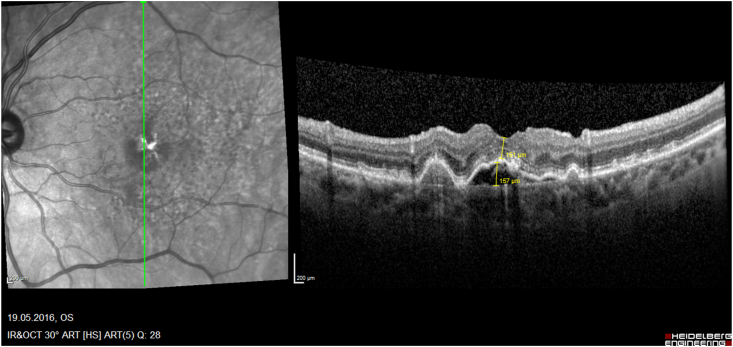
Six years postoperative status of a PED with a reduced heights of 157 μm, a fully closured the macular hole, retinal thickness 151 μm.

**Fig. 2e:**
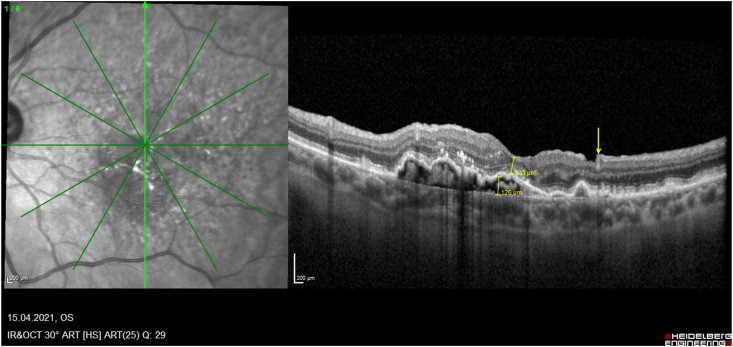
Ten years postoperatively, there is an extension of the PED while the heights of 126 μm remains reduced, a fully closured the macular hole, retinal thickness 133 μm. The yellow error indicates the still visible edge of the ILM-peeling. (For interpretation of the references to colour in this figure legend, the reader is referred to the Web version of this article.)

Our findings correlate with most of the published cases 7, 29, 30, 31, 32, 33, 34, 35, 36: most surgeons determined a small MH on the preoperative OCT, with frequent signs of ERM or VMT, undermining vitreous tractions as a causative factor. They also predominantly performed a PPV with a consecutive ILM-peel and gas tamponade. All but one MH closed, and no surgeon reported an RPE-tear formation or SR haemorrhage intra- or post-operatively. Thus, overall, the detached RPE-monolayer appears to be strong and elastic enough to resist an ILM-peeling. No MH demonstrated a late reopening after PPV. All the treated eyes experienced some functional improvement up to the best corrected VA prior to the MH-formation. These are important information encouraging a surgical treatment of a MH on top of a PED.

## 4 Conclusions

The treatment of eyes with PED and VMT or ERM under anti-VEGF injections may, in rare cases, induce a small but full thickness MH-formation. These rare sequelae of occult MH are frequently associated with an unexplained decrease in central vision, requiring intense raster OCT-evaluations. Our case is complementary to others describing the surgical closure of full thickness MHs on PED-lesions after PPV with ILM-peeling. Treatment remains challenging but appears to be safe if caution is considered.

## Authorship

All authors state that they meet the current ICMJE criteria for Authorship.

## Authors’ contributions

All authors read and approved the final manuscript.

## Funding

There was no funding or grant support.

## Patient consent

Consent to publish the case report was obtained. This report does not contain any personal information that could lead to the identification of the patient.

## Sample credit author statement

**Paula S Meyer:** Original draft preparation, Image collection, Formal analysis, Writing-, **Marc T Kammann**: Reviewing, Editing and Visualization, **Carsten H. Meyer**: Image analysis, Conceptualization, Methodology, Supervision, Reviewing.

## Declaration of competing interest

All authors have no financial disclosures.
